# Prion Protein Is Decreased in Alzheimer's Brain and Inversely Correlates with BACE1 Activity, Amyloid-β Levels and Braak Stage

**DOI:** 10.1371/journal.pone.0059554

**Published:** 2013-04-05

**Authors:** Isobel J. Whitehouse, J. Scott Miners, Elizabeth B. C. Glennon, Patrick G. Kehoe, Seth Love, Katherine A. B. Kellett, Nigel M. Hooper

**Affiliations:** 1 School of Molecular and Cellular Biology, Faculty of Biological Sciences, University of Leeds, Leeds, United Kingdom; 2 Dementia Research Group, School of Clinical Sciences, Institute of Clinical Neurosciences, University of Bristol, Bristol, United Kingdom; INSERM, UMR-S747, France

## Abstract

The cellular prion protein (PrP^C^) has been implicated in the development of Alzheimer's disease (AD). PrP^C^ decreases amyloid-β (Aβ) production, which is involved in AD pathogenesis, by inhibiting β-secretase (BACE1) activity. Contactin 5 (CNTN5) has also been implicated in the development of AD by a genome-wide association study. Here we measured PrP^C^ and CNTN5 in frontal cortex samples from 24 sporadic AD and 24 age-matched control brains and correlated the expression of these proteins with markers of AD. PrP^C^ was decreased in sporadic AD compared to controls (by 49%, p = 0.014) but there was no difference in CNTN5 between sporadic AD and controls (p = 0.217). PrP^C^ significantly inversely correlated with BACE1 activity (r_s_ = −0.358, p = 0.006), Aβ load (r_s_ = −0.456, p = 0.001), soluble Aβ (r_s_ = −0.283, p = 0.026) and insoluble Aβ (r_s_ = −0.353, p = 0.007) and PrP^C^ also significantly inversely correlated with the stage of disease, as indicated by Braak tangle stage (r_s_ = −0.377, p = 0.007). CNTN5 did not correlate with Aβ load (r_s_ = 0.040, p = 0.393), soluble Aβ (r_s_ = 0.113, p = 0.223) or insoluble Aβ (r_s_ = 0.169, p = 0.125). PrP^C^ was also measured in frontal cortex samples from 9 Down's syndrome (DS) and 8 age-matched control brains. In contrast to sporadic AD, there was no difference in PrP^C^ in the DS brains compared to controls (p = 0.625). These data are consistent with a role for PrP^C^ in regulating Aβ production and indicate that brain PrP^C^ level may be important in influencing the onset and progression of sporadic AD.

## Introduction

Alzheimer's disease (AD) is the most common form of dementia and its socioeconomic impact is increasing as the population ages [1]. The number of individuals suffering from AD worldwide is predicted to rise to 34 million by 2025 [2]. AD is characterised pathologically by the formation of intracellular neurofibrillary tangles and extracellular amyloid plaques. Neurofibrillary tangles, composed of hyperphosphorylated and aggregated tau [3], initially appear in the entorhinal cortex and hippocampus, before the spread of tau pathology into other regions [4]. Tau pathology is staged in AD using the Braak system, encompassing 6 stages which are distinguished according to the distribution of neurofibrillary tangles [4]. As tau pathology spreads, it is accompanied by neuronal loss, following which the tau may be found in the extracellular space – either in a monomeric form or in an aggregated form where it is assembled in extracellular ghost tangles [5]. Amyloid plaques are composed of the amyloid-β peptide (Aβ). Aβ is derived from the sequential cleavage of the amyloid-β precursor protein (APP) first by the β-secretase, β-site APP cleaving enzyme-1 (BACE1), and then by the γ-secretase complex. A number of rare autosomal dominant mutations in the genes encoding either APP or components of the γ-secretase complex have been identified which cause early-onset, or familial, AD. The majority of AD patients, however, do not have such underlying genetic factors and, although some risk factors have been identified (e.g. ageing and the ε4 allele of the apolipoprotein E gene), the cause of these sporadic AD cases remains unknown.

Relatively little is known about the physiological roles of APP, Aβ and BACE1; several studies have endeavoured to investigate the normal biology of these proteins and to identify other interacting proteins which may be involved in their regulation, trafficking and processing. A study of the APP interactome [6] identified several potential APP-interacting proteins, one of which was from the contactin family of proteins, and a later genome-wide association study (GWAS) identified contactin 5 (CNTN5) as one of 13 genes that showed an association with AD [7]. CNTN5 has also been associated with AD neuroimaging measures such as white matter lesion volume and entorhinal cortex thickness [8]; however, the amount of CNTN5 in the AD or ageing brain has not been reported previously. An even greater effort has been made to establish the proteins interacting with BACE1 as it is the BACE1 cleavage of APP that is the rate-limiting step in Aβ production [9], and BACE1 is a potential therapeutic target for AD. BACE1 activity in the brain is increased in sporadic AD and correlates with increased Aβ load [10], [11], [12], indicating a disruption in the normal regulation of BACE1 activity. Several proteins regulating BACE1 activity have been identified [13], [14], including the cellular form of the prion protein (PrP^C^) [15]. PrP^C^ inhibited the action of BACE1 towards wild type human APP in cellular models and the levels of endogenous murine Aβ were significantly increased in the brain of PrP^C^ null mice [15], and we proposed that a normal function of PrP^C^ may be to protect against AD [16], i.e. that BACE1 activity is negatively modulated by PrP^C^, which thereby influences Aβ load and the onset and severity of AD. Consistent with this hypothesis, we reported that, in a small cohort, PrP^C^ was decreased in the hippocampus in sporadic AD [17], although we did not examine the relationship to BACE1 activity and Aβ load.

In this study we measured PrP^C^ and CNTN5 in frontal neocortex from cases of sporadic AD and age-matched control brain samples. We confirmed our previous finding [17], in a new, larger patient cohort, that PrP^C^ is decreased in sporadic AD and demonstrate that CNTN5 levels are unchanged in sporadic AD. As PrP^C^ is decreased [17], and BACE1 activity is increased, in sporadic AD [10], [18], and as PrP^C^ negatively modulates BACE1 activity [15], [19], we tested the hypothesis that there is a negative correlation between PrP^C^ and (i) BACE1 activity, (ii) Aβ and (iii) Braak tangle stage, in human brain tissue. We found that PrP^C^ did indeed correlate inversely with BACE1 activity, Aβ load, soluble and insoluble Aβ levels, and with the severity of disease, as measured by Braak tangle stage. CNTN5, however, showed no correlation with Aβ load, soluble or insoluble Aβ level.

We previously showed that while PrP^C^ is decreased in sporadic AD and also declines with age, there is no alteration in PrP^C^ in familial AD cases [17]. Down's syndrome (DS) is caused by an extra copy of chromosome 21, which results in development abnormalities and neuropathology in the brain that are similar to that seen in AD. APP maps to chromosome 21 and trisomy 21 results in increased APP and Aβ production and early senile plaque formation [20]. Here we demonstrate that PrP^C^ levels are unchanged in the cortex in DS, compared to age-matched controls.

## Results

### PrP^C^ is reduced but CNTN5 is unchanged in sporadic Alzheimer's disease

**Figure 1:**
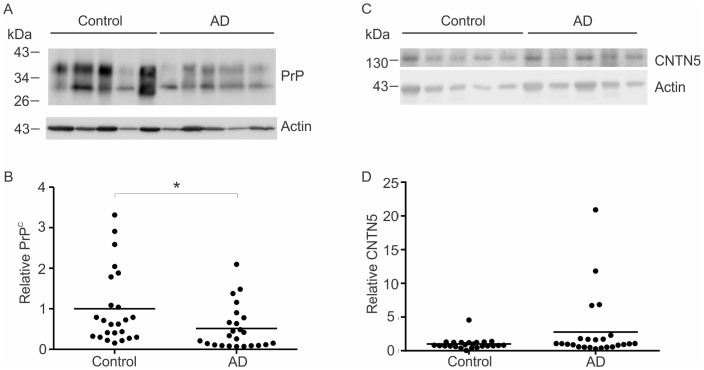
PrP^C^, but not CNTN5, is decreased in sporadic AD. Representative immunoblots of PrP^C^ and actin in temporal cortex samples from sporadic AD patients compared to age-matched controls (A) with densitometric analysis relative to actin levels represented in a grouped scatter plot (B). Representative immunoblots of CNTN5 relative to actin (C) with densitometry analysis (D). Line represents mean, *p<0.05, n = 24 per group.

Quantitative immunoblotting was used to assess PrP^C^ and CNTN5 in the temporal cortex from sporadic AD individuals in comparison to that in the brain of age-matched cognitively normal individuals. PrP^C^ was significantly reduced in sporadic AD by a mean of 49% (p = 0.014) compared to the age matched controls (Figure 1A and B, Table 1) but there was no significant difference in CNTN5 between sporadic AD and controls (Figure 1C and D, Table 1). PrP^C^ is variably glycosylated at two asparagine residues (N181 and N197), so the protein appears on immunoblots as multiple bands corresponding to un-, mono- and diglycosylated species [21]. We previously reported that PrP^C^ declines with age in the human brain [17] but there was no significant difference in age between the sporadic AD and control cases (mean age ± SEM; 82.5±1.4 years and 76.5±2.7 years, respectively, p = 0.204) (Table 1 and Table S1), indicating that the reduction of PrP^C^ in individuals with sporadic AD cannot simply be attributed to the effects of age. To ensure age had no effect, the three youngest controls (43, 48 and 53 years) were omitted to give a control mean age of 80.5±1.7 years. PrP^C^ was still significantly reduced in sporadic AD by a mean of 41% (p = 0.032) compared to age matched controls (Figure S1). In addition, there was no significant difference in the level of neuron-specific enolase (NSE) between the sporadic AD and control samples (Table 1), indicating that the lower PrP^C^ in the sporadic AD samples was not caused by neuronal loss. The post-mortem delay was also not significantly different between the sporadic AD and control group (Table 1 and Table S1).

**Table 1:** Summary data of the sporadic AD and age-matched control samples.

Characteristic	Control (n = 24*)	AD (n = 24)	p value
Age (y)	76.46±2.72	82.50±1.44	0.204
PM delay (h)	38.50±6.27	51.08±4.11	0.100
APOE ε4 allele (%)	13%	75%	<0.001
NSE (pg/ml)	0.51±0.05	0.51±0.05	0.992
PrP^C^ protein (arbitrary units)	1.00±0.19	0.51±0.11	0.014
CNTN5 protein (arbitrary units)	1.00±0.17	2.78±0.96	0.167
BACE1 activity (relative fluorescence units)	2314.79±133.28	2720.45±169.71	0.066
Soluble Aβ (nM)	1.62±0.36	2.54±0.71	0.391
Insoluble Aβ (nM)	20.44±6.56	138.92±16.41	<0.001
Aβ load (%)	0.04±0.04	2.42±0.59	<0.001
Braak stage	1.33±0.23	5.08±0.16	<0.001

All data are mean ± SEM unless stated. *n = 20 for control Aβ load analysis.

### PrP^C^ levels inversely correlate with BACE1 activity, Aβ load and Braak stage

**Figure 2:**
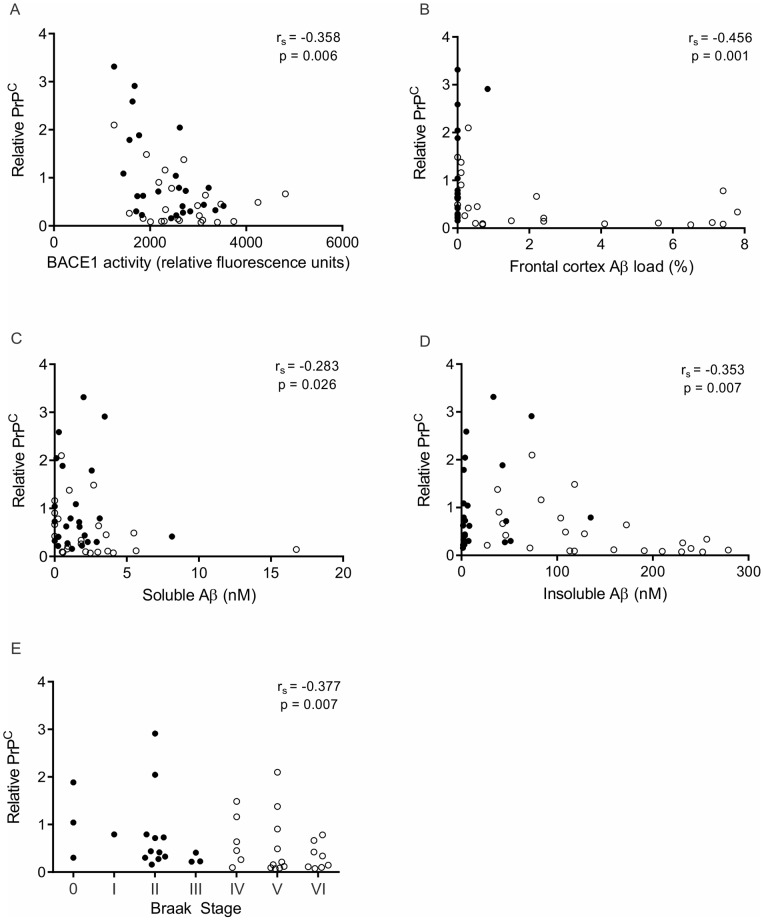
PrP^C^ inversely correlates with BACE1 activity, Aβ load, soluble and insoluble Aβ and Braak stage. Relative PrP^C^ protein levels were plotted against BACE1 activity (A), Aβ load (B), soluble Aβ (C), insoluble Aβ (D) and Braak stage (E) for each subject in the cohort (n = 48. Control, filled circles; AD, empty circles). PrP^C^ significantly inversely correlates with BACE1 activity, Aβ load, soluble Aβ, insoluble Aβ and Braak stage as determined by Spearman's rank correlation coefficient (r_s_).

As PrP^C^ negatively regulates the activity of BACE1 towards APP [15], [19], we investigated whether there was a correlation between PrP^C^, measured by immunoblotting, and BACE1 activity, measured using a fluorogenic peptide substrate (Table 1). Across the cohort there was a statistically significant inverse correlation between PrP^C^ and BACE1 activity (Figure 2A) (r_s_ = −0.358, p = 0.006), consistent with PrP^C^ normally acting to inhibit BACE1. We next examined whether the negative modulation of BACE1 activity by PrP^C^ influenced the Aβ plaque load in an individual. To do this we analysed frontal cortex Aβ levels by immunohistochemical staining of Aβ and measurement of both soluble and insoluble Aβ levels by ELISA and then correlated these data with PrP^C^. The Aβ plaque load, as determined by immunohistochemical staining was significantly higher in AD than controls (Table 1) and, in addition, significantly inversely correlated with PrP^C^ (Figure 2B) (r_s_ = −0.456, p = 0.001). Soluble Aβ levels were not statistically different between AD and controls (Table 1), but soluble Aβ did significantly inversely correlate with PrP^C^ (Figure 2C) (r_s_ = −0.283, p = 0.026). Insoluble Aβ was significantly higher in AD compared with controls (Table 1) and significantly inversely correlated with PrP^C^ (Figure 2D) (r_s_ = −0.353, p = 0.007). Finally, as PrP^C^ correlated inversely with both BACE1 and Aβ load, we went on to examine whether PrP^C^ correlated with disease severity, as measured by Braak stage (Table 1). This analysis revealed a statistically significant inverse correlation between PrP^C^ and Braak stage (r_s_ = −0.377, p = 0.007) across the cohort (Figure 2E). Again, to ensure age had no effect on the outcome, all correlation analyses were also carried out omitting the three youngest controls (43, 48 and 53 years). PrP^C^ remained inversely correlated with BACE1 activity, Aβ levels and Braak stage (data not shown).

### CNTN5 levels do not correlate with soluble or insoluble Aβ

**Figure 3:**
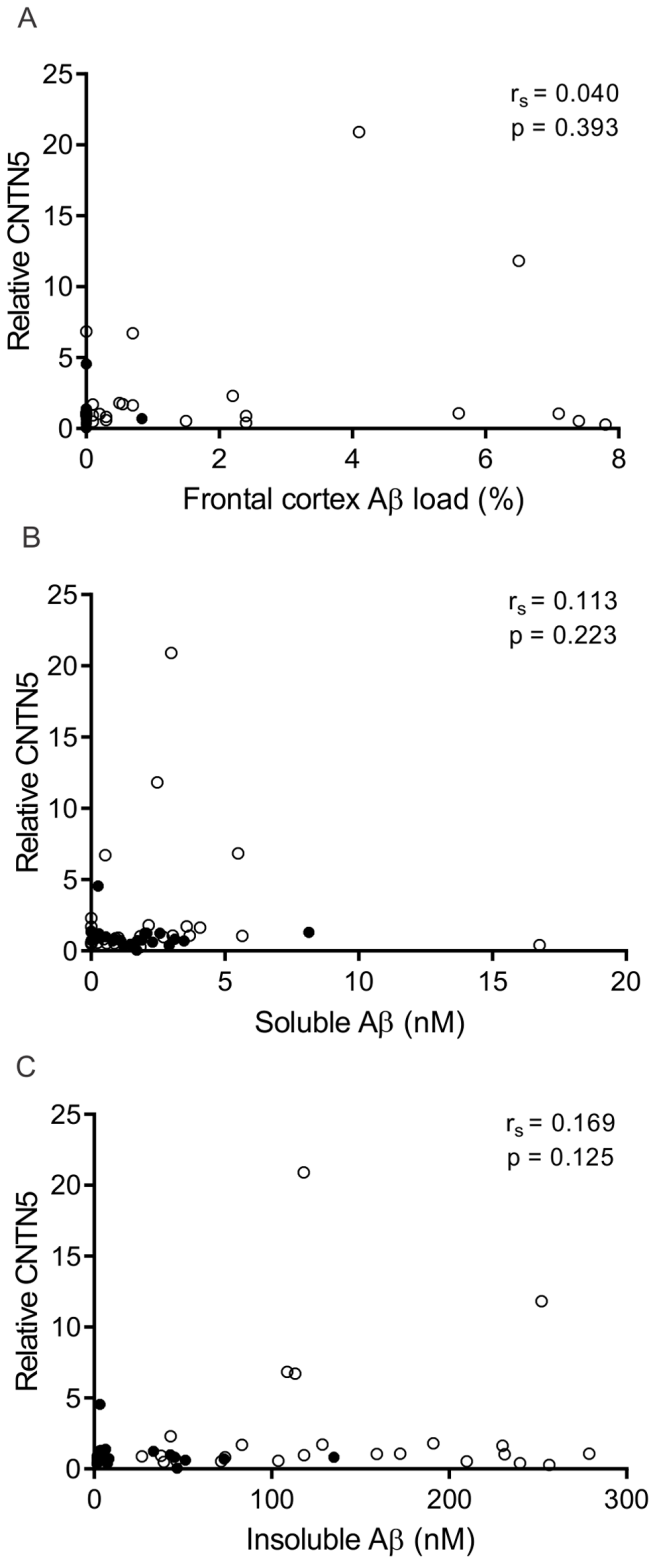
CNTN5 does not correlate with Aβ load, soluble Aβ or insoluble Aβ. Relative CNTN5 protein levels were plotted against Aβ load (A), soluble Aβ (B) and insoluble Aβ (C) for each subject in the cohort (n = 48. Control, filled circles; AD, empty circles). CNTN5 levels did not correlate with Aβ load, soluble Aβ or insoluble Aβ levels as determined by Spearman's rank correlation coefficient (r_s_).

Although CNTN5 levels are unchanged in sporadic AD this does not rule out a correlation of this protein with markers of disease progression. As CNTN5 has been identified as having an association with AD by GWAS [7], we also examined the relation between CNTN5 and Aβ load. CNTN5 did not correlate with Aβ load (Figure 3A), soluble Aβ (Figure 3B) or insoluble Aβ (Figure 3C).

### PrP^C^ is not reduced in Down's syndrome

**Figure 4:**
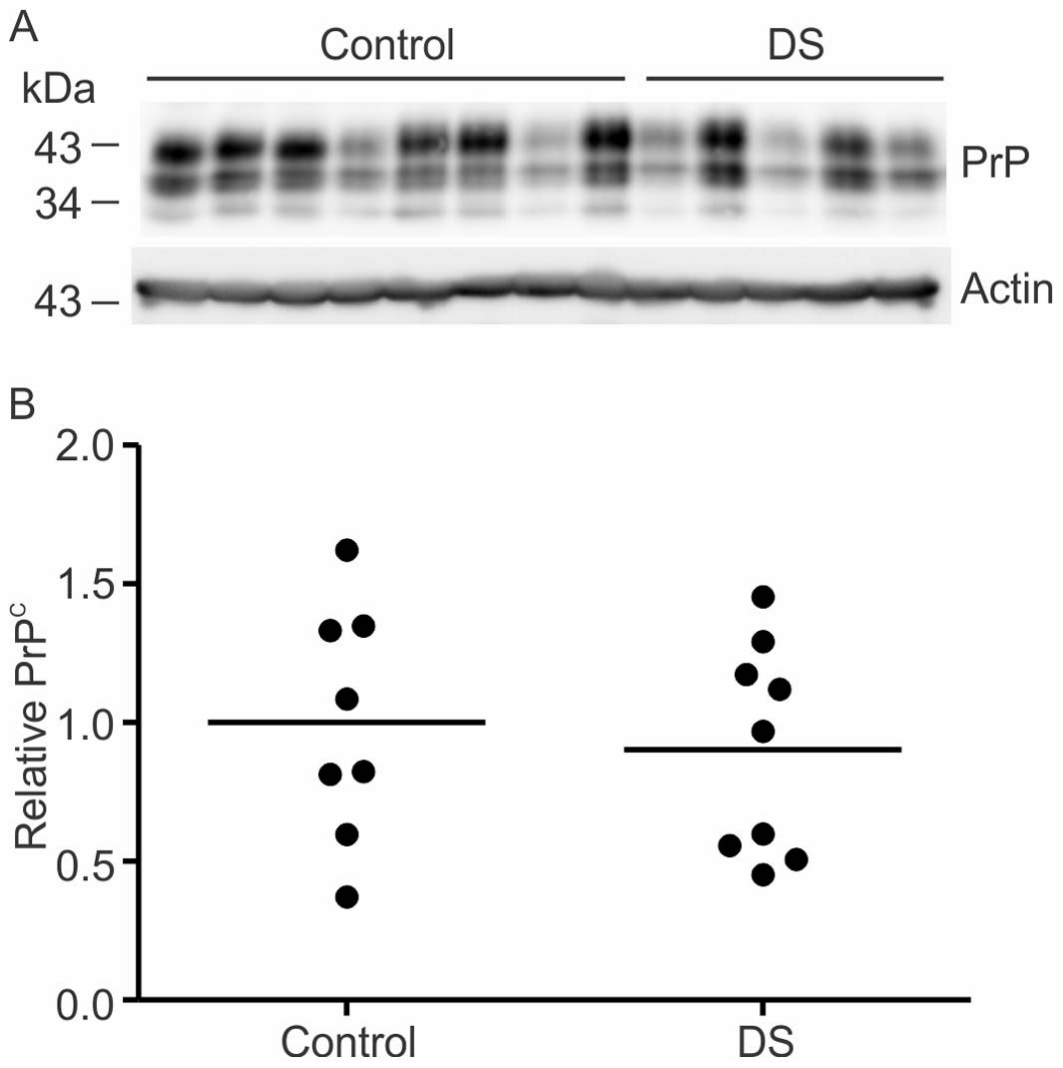
PrP^C^ is not reduced in DS brains. Representative immunoblots of PrP^C^ and actin in temporal cortex samples from Down's syndrome patients compared to age-matched controls (A). Densitometric analysis of PrP^C^ relative to actin levels is represented in grouped scatter plot (B). Line represents mean, *p<0.05, n = 8 control group and n = 9 DS group.

PrP^C^ was also measured in frontal cortex samples from DS and control brains. Frontal cortex samples were immunoblotted for PrP^C^ and actin (Figure 4A and B). PrP^C^ was not significantly different in the DS compared to the control brains (Figure 4B and Table 2). BACE1 activity and Aβ levels were also assessed in the DS and control cohort. BACE1 activity, although higher in the DS brains, did not differ significantly from control values (p = 0.061, Table 2). Soluble Aβ level, although higher in the DS brain, was not significantly different from controls (p = 0.179, Table 2). Insoluble Aβ, however, was significantly increased in the DS brain compared to controls (p<0.001, Table 2). The DS and control cohorts were closely matched in age (p = 0.226) (Table 2 and Table S2). The post-mortem delay was not significantly different between the DS and control groups (p = 0.217) (Table 2 and Table S2).

**Table 2:** Summary data of the DS and age-matched control samples.

Characteristic	Control (n = 8)	DS (n = 9)	p value
Age (y)	62.63±3.58	59.89±2.18	0.236
PM delay (h)	26.50±6.98	39.22±6.92	0.217
PrP^C^ protein (arbitrary units)	1.00±0.15	0.90±0.13	0.625
BACE1 activity (relative fluorescence units)	1173.46±75.61	1429.78±98.18	0.061
Soluble Aβ (nM)	4.78±0.42	7.32±1.69	0.179
Insoluble Aβ (nM)	22.38±15.36	288.84±36.82	<0.001

All data are mean ± SEM.

## Discussion

In this study we have demonstrated a significant inverse correlation between PrP^C^ and BACE1 activity in cortex from patients with sporadic AD (in whom PrP^C^ level is lower and BACE1 activity higher than in age-matched controls) but not in Down's syndrome, despite the accumulation of Aβ and the presence of other AD-type pathological abnormalities in the latter. We previously demonstrated that PrP^C^ negatively modulates the activity of BACE1 [15], in part through an interaction of PrP^C^ with the prodomain of the immature form of BACE1 within the Golgi, thereby decreasing the amount of BACE1 that is trafficked to the cell surface and endosomes where it cleaves wild type APP [19]. To test our previous hypothesis [16] that PrP^C^ may function normally to protect against AD by reducing BACE1 activity, we have explored the relationship between PrP^C^ level and AD pathology in two contexts: first in sporadic AD, and second in Down's syndrome. The latter group provides an opportunity to explore another Aβ-related condition since people with DS also develop abundant AD pathology, but this is attributable to increased production of APP. Previous work has demonstrated that BACE1 activity in the brain increases with age and in association with Aβ accumulation [10], [12], [18], [22], although the molecular mechanisms underlying this are unclear.

As PrP^C^ is a negative modulator of BACE1 activity, we hypothesised that it would significantly impact on Aβ levels. Our data revealed a significant inverse relationship between PrP^C^ and both soluble and insoluble Aβ as well as between PrP^C^ and Aβ plaque load, indicating that the relationship between PrP^C^ and BACE1 may have important downstream effects on the development of AD. In addition, we found a significant inverse correlation between PrP^C^ and Braak stage, a marker of disease severity or progression. The correlation with Braak stage is likely to be an indirect indicator of the influence of PrP^C^ on AD progression, as there are no data to support a direct role for PrP^C^ in preventing neurofibrillary tangle formation. However, taken together these results indicate that PrP^C^ levels in the brain may be an important factor influencing not only the onset but also the progression of sporadic AD.

Importantly, the correlations extended across the entire cohort (both AD cases and age-matched controls). There was a significant inverse correlation between PrP^C^ and BACE1 activity, Aβ load, soluble and insoluble Aβ levels and Braak stage, independent of the clinical diagnosis of AD. The symptoms of AD typically progress from mild symptoms of memory loss to severe dementia and it has been suggested that impairment in multiple cognitive domains is observable several years before a clinical diagnosis of AD is made [23]. This observed cognitive dysfunction is not qualitatively different from that seen in normal ageing, and a continuum from normal ageing to preclinical dementia has been proposed [24]. We showed previously that PrP^C^ decreases with age in the brain [17] and our current data suggest that an inverse correlation between PrP^C^ and BACE1 activity may anticipate the onset of sporadic AD. Taken together, these findings point towards a decrease in PrP^C^ in the brain as a primary contributor to the development of disease, at least in some cases of sporadic AD. In addition, the data suggest that the level of PrP^C^ in the brain may be critical in determining the onset and progression of sporadic AD through its modulation of BACE1 activity.

Down's syndrome (DS) is caused by an extra copy of chromosome 21, which results in developmental abnormalities and also neuropathology in the brain that is similar to that in AD. APP maps to chromosome 21 and trisomy 21 results in increased APP and Aβ production and early plaque formation [20]. Here we demonstrate that the level of PrP^C^ is unchanged in the cortex in DS, compared to age-matched controls, confirming that the change in PrP^C^ in sporadic AD is not a secondary consequence of disease. Previous work has implicated the APP intracellular domain (AICD) as a transcription factor regulating PrP^C^ expression, suggesting that over-expression of APP may increase PrP^C^ expression [25]. However, in multiple cell lines and two transgenic mouse lines expressing human APP, we could find no evidence for APP-mediated regulation of the expression of PrP^C^ [26]. Here we have demonstrated that PrP^C^ is unchanged in brains from DS patients, indicating that the over-expression of APP does not alter PrP^C^ expression in the human brain. A recent study reported that PrP^C^ is unchanged in the AD brain [27]. However, while the cohort was well characterised in terms of diagnosis, the authors did not provide any information as to whether the AD cases were familial or sporadic. Previously we reported that PrP^C^ level was unchanged in the brain in familial AD cases (with mutations in either APP or presenilin-1) [17], indicating that differentiating between the two forms of AD is crucial in evaluating any changes in PrP^C^. The decrease in PrP^C^ protein that we have observed in sporadic AD would be consistent with a recent report of decreased *PRNP* mRNA in AD patients [28].

We have also demonstrated that CNTN5, a protein thought to interact with APP and identified through GWAS [7] as being associated with AD, is unchanged in sporadic AD and does not correlate with Aβ load, soluble Aβ or insoluble Aβ in our cohort. If CNTN5 has a role in the development of AD it appears not to be related to CNTN5 expression level. CNTN5 may, however, contribute to the development of AD without any alteration in its expression level. The subcellular locations of contactin proteins are tightly regulated by their post-translational processing and interactions with contactin-associated proteins [29], [30], [31]. Cellular trafficking and therefore the subcellular location of CNTN5 may be altered in AD.

Recently, we reported that PrP^C^ mediates the uptake of extracellular zinc into neuronal cells [32]. Zinc promotes the aggregation of Aβ into toxic oligomeric forms [33] and in an AD mouse model, synaptic zinc was shown to increase insoluble Aβ and its deposition in plaques [34]. In addition, synaptic zinc favours the attachment of Aβ oligomers to the N-methyl-D-aspartate (NMDA) receptor, mediating their excitotoxicity [35]. The reduction in PrP^C^ in the brain in sporadic AD would be expected to result in decreased zinc uptake. This may result in an increase in the amount of zinc in the synaptic cleft which would promote Aβ aggregation and synaptic targeting, potentially contributing also to the neurodegenerative process in AD.

In conclusion, our data demonstrate that the level of PrP^C^ is inversely correlated with BACE1 activity and Aβ in the human brain. These findings implicate changes in PrP^C^ in the pathogenesis of sporadic AD and suggest that modulating PrP^C^ level may have an impact on the development and course of sporadic AD.

## Materials and Methods

### Ethics statement

Brain tissue was obtained from the South West Dementia Brain Bank, University of Bristol, UK. The study was conducted with approval from the North Somerset and South Bristol Research Ethics Committee and the Leeds Central Research Ethics Committee.

### Study cohorts

All cases had been subjected to detailed neuropathological examination. AD cases had been assessed according to the criteria of the Consortium to Establish a Registry for Alzheimer's Disease (CERAD) [36]. All DS cases had been confirmed genetically. The controls had no history of cognitive decline or dementia, showing the absence of AD (as defined by CERAD) or other neuropathological abnormalities. Total soluble and total guanidine-extracted Aβ levels [37], [38], Aβ plaque load [39], [40], BACE-1 activity [12], [22], and NSE levels [41] had previously been measured in all cases which had also previously been categorised according to the Braak tangle stage [41]. The AD, DS and control groups were matched for post-mortem delay, age-at-death, and gender as presented in Tables 1, 2, S1 and S2.

### Tissue Preparation

For measurements of BACE-1 activity, PrP^C^ protein and NSE, approximately 200 mg of frontal neocortex (Brodmann area 6) was homogenised in 1 ml lysis buffer (0.5% Triton X-100, 20 mM Tris/HCl pH 7.4, 10% (wt/vol) sucrose containing aprotinin (1 µg/ml) and phenylmethane sulfonyl fluoride (PMSF; 10 µM)) (all reagents from Sigma Aldrich, Poole, UK). Brain tissue was homogenized for 30 seconds in a Precellys 24 automated tissue homogenizer (Stretton Scientific, Derbyshire, UK) with 2.3-mm silica beads (Biospec, Thistle Scientific, Glasgow, UK) and total protein was measured using Total Protein kit (Sigma Aldrich). The homogenates were centrifuged at 20 17 *g* for 15 min at 4°C, and aliquots of the supernatant were stored at −80°C until used.

For measurements of Aβ, tissue (200 mg) was allowed to thaw to 4°C, homogenised in 5 volumes (wt: vol) of Tris-buffered saline (TBS) extraction buffer [140 mM NaCl, 3 mM KCl, 25 mM Tris/HCl, pH 7.4, containing 1% Nonidet P-40 (NP40), 5 mM EDTA, 2 mM 1,10-phenanthroline, 10 µM PMSF and 1 µg/ml aprotinin (all reagents from Sigma Aldrich), as detailed in [37], [38]. The homogenate was then centrifuged at 20 817 *g* for 15 min at 4 C and the supernatant (soluble fraction) was stored at −80 C until used. The pellet was homogenized in 6.25 M guanidine HCl in 50 mM Tris/HCl, pH 8.0, incubated for 4 h at 25 C and centrifuged at 20 817 *g* for 20 min at 4°C. The resultant supernatant (guanidine-extractable fraction) was stored at −80°C until used.

### Immunoblotting of PrP^C^ and CNTN5

Samples were mixed with an equal volume of SDS dissociation buffer (125 mM Tris/HCl, pH 6.8, 2% (w/v) SDS, 20% (v/v) glycerol, 100 mM dithiothreitol, 0.002% (w/v) bromophenol blue), and boiled for 5 min. Proteins were resolved by SDS polyacrylamide gel electrophoresis using 10% (CNTN5) and 14.5% (PrP^C^) polyacrylamide gels. Resolved proteins were transferred to Immobilon P polyvinylidene difluoride membrane (Amersham, Little Chalfont, UK). The membrane was blocked by incubation for 1 h with PBS containing 0.1% (v/v) Tween-20 and 5% (w/v) dried milk powder. Antibody incubations were performed in PBS Tween containing 2% (v/v) bovine serum albumin. Antibody 6D11 (Eurogentec Ltd.) which recognises amino acids 93–109 of human PrP^C^ was used at 1∶5000, antibody AF3030 (R&D Systems, Abingdon, UK) against CNTN5 was used at 1∶500 and anti-actin antibody AC15 (Sigma, Poole, UK) was used at 1∶5000. Horseradish peroxidase-conjugated secondary antibody was used at 1∶4000 in the same buffer. Bound antibody was detected using the enhanced chemiluminescence detection method (Amersham Biosciences, Amersham, UK). Blots were stripped using 100 mM glycine, pH 2.5 for 30 min, blocked by incubation for 1 h with PBS containing 0.1% (v/v) Tween20 and 5% (w/v) dried milk powder, and reprobed using the anti-actin antibody as described above.

### Measurement of BACE-1 activity

The fluorogenic substrate (Mca-SEVNLDAEFRK(Dnp)RR-NH_2_) containing the Swedish double point mutation of APP (R&D Systems) was used according to the manufacturer's guidelines to measure BACE-1 activity (relative fluorescence units) in brain homogenates as previously reported [12], [22]. Each homogenate was assayed in duplicate in the presence and absence of the BACE1 inhibitor III (5 μM) (Millipore, Durham, UK). BACE-1 activity was interpolated from a standard curve generated from serial dilutions of recombinant human BACE-1 after subtraction of the inhibited from the uninhibited value. BACE-1 activity was finally adjusted according to total protein content (measured using the Total Protein Kit; Sigma).

### Measurement of total soluble and insoluble (guanidine-extractable) Aβ

The method of ELISA measurement of total soluble and insoluble Aβ was reported previously [37], [38]. Soluble and insoluble (guanidine-HCl-extractable) fractions were analysed by sandwich ELISA in which monoclonal anti-Aβ (4G8 clone, raised against amino acids 18–22; Millipore, Watford, UK) was used for the capture step and biotinylated anti-human Aβ monoclonal antibody (10H3 clone) (Thermo Fisher Scientific, Northumberland, UK) for the detection step.

### Measurement of neuron-specific enolase

NSE in brain homogenates was measured by a direct ELISA as described previously [41]. Serial dilutions of recombinant human NSE (Biomol, Exeter, UK) were used to construct a best-fit curve, and NSE concentrations were calculated by interpolation. Each sample was assayed in duplicate, and the mean was determined. The NSE concentration was used to provide a proxy measurement of the number of neurons in the tissue samples.

### Measurement of Aβ load

Parenchymal Aβ load had previously been measured in all cases [39]. The field fraction (percentage area occupied by Aβ) was measured in an unbiased selection of 10 areas of cortex covering 4 mm^2^ with the help of Histometrix software (Kinetic Imaging, Wirral, UK) driving a Leica DM microscope with a motorised stage. Aβ-laden blood vessels were excluded from analysis.

### Statistical Analysis

Densitometric analysis was performed using either the advanced image data analyser (AIDA) programme (Raytest Scientific Ltd) or Image J 1.44p (National Institutes of Health, USA). Quantification of PrP^C^ and CNTN5 was in relation to actin. The distribution of the AD cases compared to their age-matched controls was determined by the Kolmogorov-Smirnov test. Group data were compared using either an Independent T-test for parametric, or a Mann-Whitney U test (with an exact test for ApoE ε4 analysis) for non-parametric data. One-tailed Spearman's rank correlation coefficient was used to assess the correlation of PrP^C^ and CNTN5 to BACE1 activity, soluble and insoluble Aβ and Braak Stage, p≤0.05 was considered significant. The data were analysed using the Statistical Package for Social Sciencs (SPSS 17.0) program (Chicago, USA) and GraphPad Prism (version 6) (Graphpad Software Inc , California, USA).

## Supporting Information

Figure S1 **PrP^C^ is decreased in Sporadic AD.** Densitometric analysis of PrP^C^ levels relative to actin represented in a grouped scatter plot. Line represents mean, *p<0.05, n = 21 control group and n = 24 AD group.
(TIF)

Table S1 **Characteristics of the sporadic AD and control subjects used in the study.**
(DOCX)

Table S2 **Characteristics of the Down's syndrome and control subjects used in the study.**
(DOCX)
