# Supplementary figures and images for: Prion Protein Is Decreased in Alzheimer's Brain and Inversely Correlates with BACE1 Activity, Amyloid-β Levels and Braak Stage
Source: PLoS One. 2013 Apr 5;8(4):e59554. doi: 10.1371/journal.pone.0059554 (PMC3618446; doi:10.1371/journal.pone.0059554)

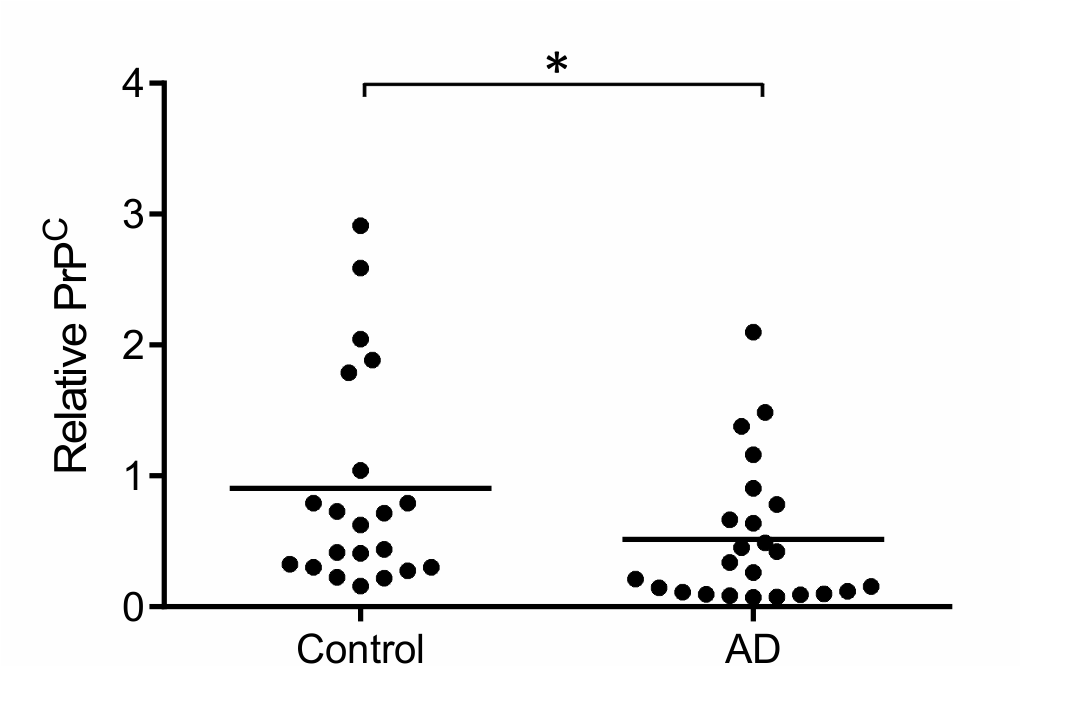

Supplement: Figure S1 — PrPC is decreased in Sporadic AD. Densitometric analysis of PrPC levels relative to actin represented in a grouped scatter plot. Line represents mean, *p<0.05, n = 21 control group and n = 24 AD group. (TIF) [file pone.0059554.s001.tif]
