# Supplementary material for: Prion Protein Is Decreased in Alzheimer's Brain and Inversely Correlates with BACE1 Activity, Amyloid-β Levels and Braak Stage
Source: PLoS One. 2013 Apr 5;8(4):e59554. doi: 10.1371/journal.pone.0059554 (PMC3618446; doi:10.1371/journal.pone.0059554)
Supplement: Table S1 — Characteristics of the sporadic AD and control subjects used in the study. (DOCX) [file pone.0059554.s002.docx]

**Table S1: Characteristics of the sporadic AD and control subjects used in the study**

| **Gender** | **Age (y)** | | **APOE ε4 allele** | **PM delay (h)** |
| --- | --- | --- | --- | --- |
| **Sporadic AD** | |  |  |  |
| M | 69 | | 4.4 | 48 |
| F | 70 | | 3.4 | 25 |
| M | 74 | | 3.4 | 50 |
| F | 74 | | 4.4 | 53 |
| F | 77 | | 3.4 | 43 |
| F | 78 | | 4.4 | 77 |
| F | 78 | | 3.4 | 9 |
| F | 78 | | 3.3 | 35 |
| M | 79 | | 3.4 | 28 |
| M | 80 | | 3.4 | 31 |
| F | 81 | | 3.4 | 42 |
| F | 81 | | 3.3 | 66 |
| F | 83 | | 3.4 | 43 |
| M | 85 | | 3.4 | 58 |
| M | 85 | | 3.4 | 66 |
| F | 87 | | 3.4 | 72 |
| F | 87 | | 2.4 | 67 |
| F | 88 | | 3.3 | 79 |
| F | 89 | | 3.3 | 71 |
| F | 89 | | 3.3 | 82 |
| F | 90 | | 4.4 | 21 |
| F | 91 | | 3.4 | 37 |
| F | 91 | | 2.4 | 70 |
| F | 96 | | 2.3 | 53 |
|  |  | |  |  |
| **Control** |  | |  |  |
| F | 43 | | - | 12 |
| F | 48 | | 2.4 | 79 |
| M | 53 | | 3.3 | 7 |
| M | 62 | | 3.4 | 4 |
| M | 64 | | 3.3 | 23 |
| F | 72 | | 3.3 | 24 |
| M | 73 | | 3.3 | 36 |
| M | 77 | | 2.3 | 10 |
| M | 78 | | 3.3 | 12 |
| M | 79 | | 3.3 | 24 |
| M | 80 | | 3.3 | 106 |
| F | 80 | | 3.3 | 92 |
| F | 81 | | 3.3 | 103 |
| M | 82 | | 3.3 | 30 |
| M | 82 | | 3.3 | 3 |
| F | 82 | | 4.4 | 37 |
| M | 82 | | 3.3 | 56 |
| M | 84 | | 3.3 | 48 |
| F | 84 | | 2.3 | 17 |
| F | 88 | | 3.3 | 62 |
| F | 88 | | 3.3 | 28 |
| M | 90 | | 2.3 | 45 |
| M | 90 | | 3.3 | 48 |
| F | 93 | | 3.3 | 18 |
