# Supplementary material for: Prion Protein Is Decreased in Alzheimer's Brain and Inversely Correlates with BACE1 Activity, Amyloid-β Levels and Braak Stage
Source: PLoS One. 2013 Apr 5;8(4):e59554. doi: 10.1371/journal.pone.0059554 (PMC3618446; doi:10.1371/journal.pone.0059554)
Supplement: Table S2 — Characteristics of the Down's syndrome and control subjects used in the study. (DOCX) [file pone.0059554.s003.docx]

**Table S2: Characteristics of the Down’s syndrome and control subjects used in the study**

| **Gender** | **Age (y)** | | **PM delay (h)** |
| --- | --- | --- | --- |
| **DS** | |  |  |
| F | 48 | | 79 |
| F | 50 | | 43 |
| F | 59 | | 24 |
| M | 62 | | 24 |
| M | 62 | | 51 |
| F | 63 | | 51 |
| M | 64 | | 16 |
| F | 64 | | 48 |
| F | 67 | | 17 |
|  |  | |  |
| **Control** |  | |  |
| F | 43 | | 12 |
| M | 53 | | 7 |
| M | 63 | | 40 |
| M | 64 | | 16 |
| M | 64 | | 12 |
| M | 69 | | 66 |
| F | 72 | | 24 |
| M | 73 | | 35 |
|  |  | |  |
